# Synergistic Interactions of MmpL3 Inhibitors with Antitubercular Compounds *In Vitro*

**DOI:** 10.1128/AAC.02399-16

**Published:** 2017-03-24

**Authors:** Wei Li, Andrea Sanchez-Hidalgo, Victoria Jones, Vinicius Calado Nogueira de Moura, E. Jeffrey North, Mary Jackson

**Affiliations:** a Mycobacteria Research Laboratories, Department of Microbiology, Immunology and Pathology, Colorado State University, Fort Collins, Colorado, USA; b Creighton University, School of Pharmacy & Health Professions, Department of Pharmacy Sciences, Omaha, Nebraska, USA

**Keywords:** Mycobacterium, tuberculosis, MmpL3, mycolic acids, drug synergism, Mycobacterium tuberculosis

## Abstract

A number of inhibitors of the essential Mycobacterium tuberculosis mycolic acid transporter, MmpL3, are currently under development as potential novel antituberculosis agents. Using the checkerboard method to study the interaction profiles of various antituberculosis drugs or experimental compounds with two different chemotypes inhibiting this transporter (indolcarboxamides and adamantyl ureas), we showed that MmpL3 inhibitors act synergistically with rifampin, bedaquiline, clofazimine, and β-lactams.

## TEXT

**FIG 1:**
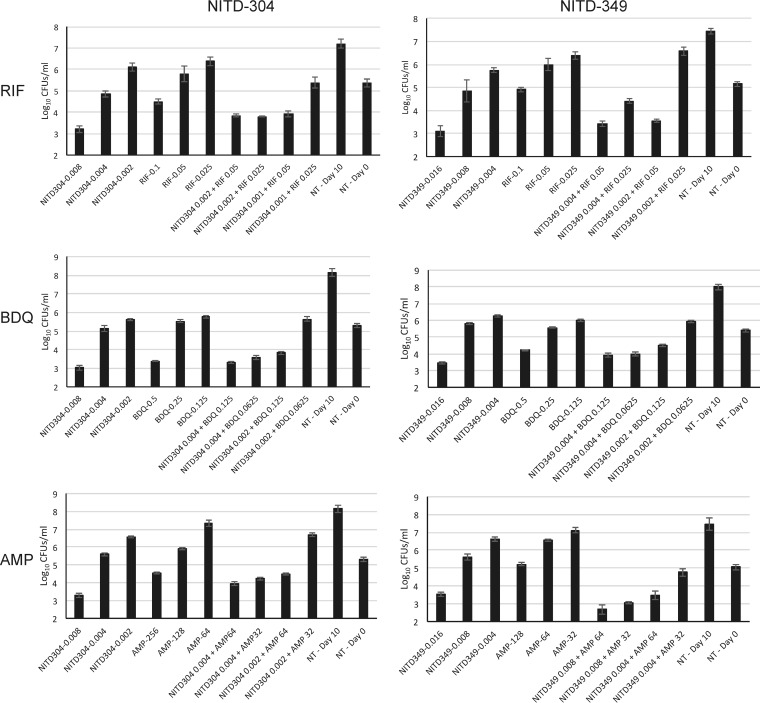

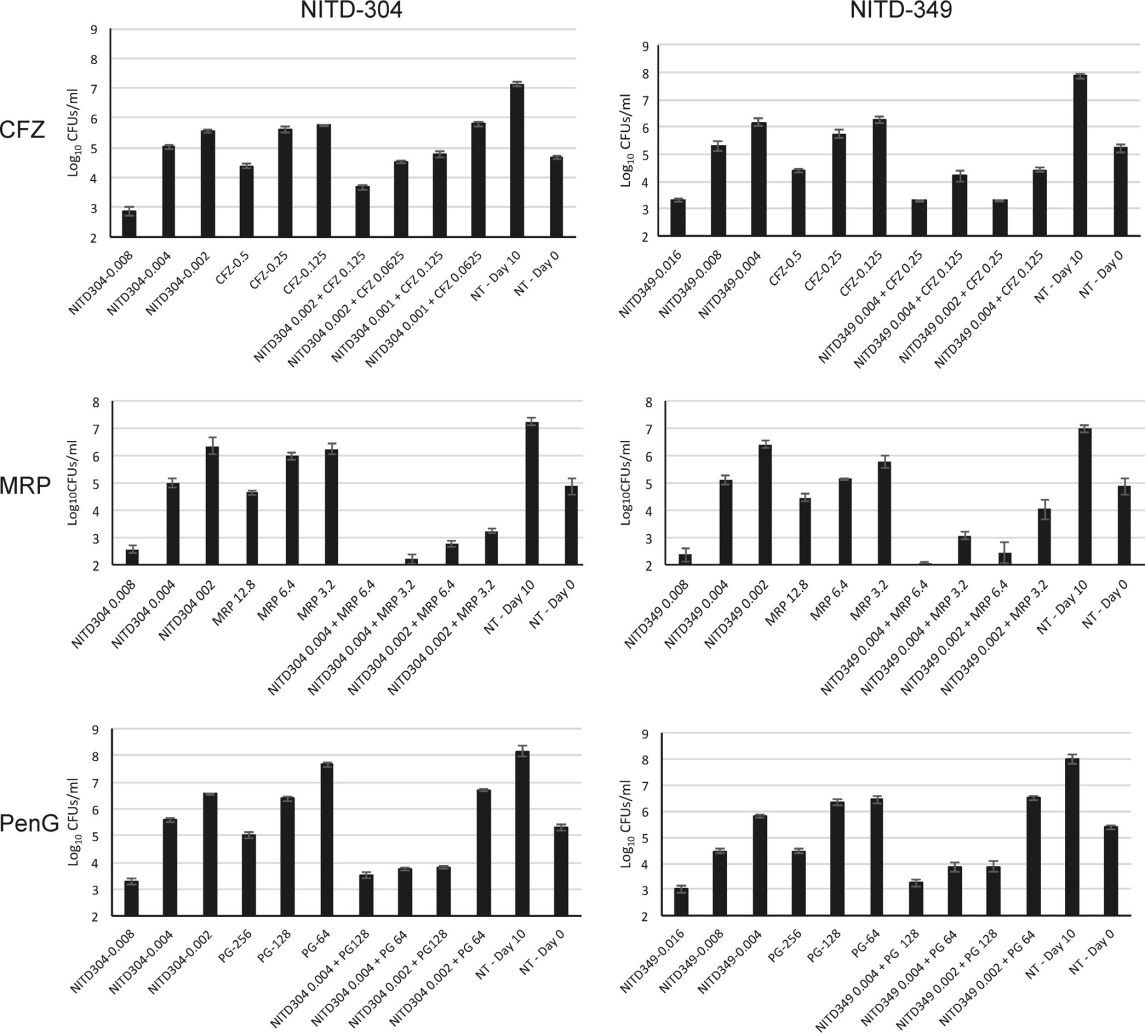
Effect of combination treatments on the viability of M. tuberculosis H37Rv mc^2^6202 as determined by CFU counts. M. tuberculosis was grown in the presence of the indicated concentrations (in micrograms per milliliter) of compounds. After 10 days of incubation at 37°C, serial dilutions of the cultures were plated on 7H11 agar to determine CFU counts. Control cultures that received no drug treatment (NT) were plated on day 0 and on day 10. The averages and standard deviations of results of triplicate CFU plating from two independent wells for each treatment condition are shown.

Better-tolerated and more-efficacious antituberculosis (anti-TB) drug regimens that meet the desired goals of decreasing treatment duration and killing multidrug-resistant Mycobacterium tuberculosis isolates while being compatible with HIV treatment are urgently needed. In recent years, the screening of compound libraries against M. tuberculosis and nontuberculous mycobacteria in culture has identified a number of novel chemical entities with potent mycobactericidal activity whose primary target appears to be the essential mycolic acid transporter MmpL3 (1–4). Among these novel chemical scaffolds, diamine- and indolamide-based compounds have emerged as particularly promising on the basis of efficacy, tolerability, and pharmacological properties (2, 5–7). The potency of these compounds is owed at least in part to the exquisite vulnerability of the MmpL3 transporter both *in vitro* and *in vivo* (1). Indeed, the inhibition of MmpL3 results in the abolition of the export of mycolic acids to the outer membrane and in the rapid killing of the bacilli (1, 8). Whether the important changes in the cell envelope composition of M. tuberculosis that follow the chemical inhibition of MmpL3 increase the efficacy of other drugs, either as a result of their increased penetration inside the bacilli or otherwise, has not been thoroughly investigated. Because of the importance of determining whether new drug candidates exhibit potential synergistic, antagonistic, or additive interactions with other antituberculosis drugs, we used the checkerboard assay (9) to investigate the *in vitro* interaction profiles of two different series of MmpL3 inhibitors with a variety of first-line and second-line anti-TB drugs and other experimental compounds, including rifampin (RIF; Sigma), isoniazid (INH; Fluka), ethambutol (EMB; Sigma), ampicillin (AMP; Sigma), penicillin G (PenG; Sigma), meropenem (MRP; Goldbio), ciprofloxacin (CIP; Fluka), bedaquiline (BDQ), and clofazimine (CFZ). The four MmpL3 inhibitors tested in this study were the indolamides NITD-304 and NITD-349, currently in preclinical development (6), and the adamantyl ureas AU1235 and AU36 (8, 10). Two-drug combinations at various concentrations below their MICs were tested for growth inhibition of M. tuberculosis in Middlebrook 7H9 broth using a two-dimensional array of 2-fold dilutions of each test compound in 96-well plates. The resazurin reduction microplate assay (REMA) was initially used as a metabolic activity readout, and the fractional inhibitory index (ΣFIC) of each drug combination was calculated as described previously (9, 11). ΣFIC values of ≤0.5 indicate synergistic activity; values of ≥4 indicate antagonism; and values between those ranges correspond to additivity (no interaction) (9). Checkerboard experiments were performed two to three times using independent culture batches, and the results were consistent between the repeats. The results, which are summarized in Table 1, revealed similar patterns of behavior among MmpL3 inhibitors regardless of the nature of their pharmacophore. Overall, all four MmpL3 inhibitors increased the susceptibility of M. tuberculosis to PenG, AMP, MRP, RIF, CFZ, and BDQ. Both indolamides displayed ΣFIC values of ≤0.5 with these six drugs, while the adamantyl ureas displayed ΣFIC values of ≤0.5, indicative of synergistic interactions against M. tuberculosis, with BDQ, MRP, and RIF. In contrast, the interaction of MmpL3 inhibitors with INH, CIP, and EMB was found to be purely additive. No antagonistic interaction was observed between any of the four MmpL3 inhibitors and the compounds tested. As expected, NITD-304 and NITD-349 showed no synergistic interaction (ΣFIC = 1). REMA results determined with NITD-304 and NITD-349 were confirmed by plating CFU to directly assess bacterial viability (Fig. 1), and the good correlation of the results of the two methods was confirmed using the paired Student *t* test (*P* < 0.0001). Wells containing NITD-304 or NITD-349 and AMP, RIF, BDQ, MRP, or CFZ at less than 4-fold their respective MIC_99_ concentrations resulted in 85% (NITD-304 plus AMP) to 99% (NITD-349 plus RIF) killing of M. tuberculosis.

**TABLE 1:** Interaction of MmpL3 inhibitors with other antimycobacterial drugs and experimental compounds against M. tuberculosis H37Rv mc^2^6206 as determined by checkerboard REMA^*a*^

Compound	MIC	Interaction with NITD-304		Interaction with NITD-349		Interaction with AU1235		Interaction with AU36	
		ΣFIC	Outcome	ΣFIC	Outcome	ΣFIC	Outcome	ΣFIC	Outcome
NITD-304	0.008								
NITD-349	0.016								
AU1235	0.2								
AU36	1								
CFZ	0.5	0.5	**Synergistic**	0.37–0.5	**Synergistic**	0.75	Additive	0.75	Additive
BDQ	0.5	0.5	**Synergistic**	0.37–0.5 *(0.5)*	**Synergistic**	0.5 *(0.37)*	**Synergistic**	0.5	**Synergistic**
AMP	256	0.5	**Synergistic**	0.5	**Synergistic**	0.75	Additive	0.5	**Synergistic**
PenG	256	0.5–0.75	**Synergistic/additive**	0.5	**Synergistic**	0.75	Additive	0.75	Additive
MRP	12.8	0.5	**Synergistic**	0.5 *(0.5)*	**Synergistic**	0.5 *(0.37)*	**Synergistic**	0.5	**Synergistic**
INH	0.04	1	Additive	1	Additive	1	Additive	1	Additive
EMB	6.4	1	Additive	0.75	Additive	0.75	Additive	0.75	Additive
RIF	0.1	0.5	**Synergistic**	0.5 *(0.5)*	**Synergistic**	0.37 *(0.37)*	**Synergistic**	0.37	**Synergistic**
CIP	0.4	1	Additive	1	Additive	1	Additive	1	Additive

a M. tuberculosis H37Rv mc^2^6206 (an avirulent Δ*panCD* Δ*leuCD* mutant of M. tuberculosis H37Rv) was grown for 10 days at 37°C in Middlebrook 7H9 broth supplemented with 10% OADC (oleic acid-albumin-dextrose-catalase) (BD, Difco), 0.5% glycerol, 0.05% tyloxapol, 0.2% Casamino Acids, 48 μg/ml pantothenate, and 50 μg/ml l-leucine before the addition of resazurin. MIC values (in micrograms per milliliter) determined by REMA were read after an additional 2-night incubation at 37°C. Six key combinations with NITD-349 and AU1235 were tested against the virulent M. tuberculosis H37Rv strain ATCC 25618 with the same results (see italicized ΣFIC values in parentheses alongside the values reported for the H37Rv mc^2^6206 strain). Boldface data indicate synergistic or synergistic/additive interactions.

**FIG 2:**
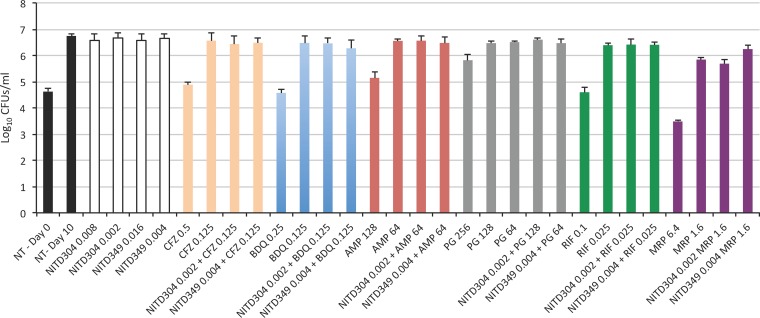
Effect of combination treatments on the viability of an indolamide- and adamantyl urea-resistant mutant of M. tuberculosis H37Rv mc^2^6202 harboring a missense mutation in MmpL3 (L567P). Compared to the results seen with wild-type M. tuberculosis H37Rv mc^2^6202 tested for susceptibility to the same drug combinations (see Fig. 1), synergistic interactions were lost in the mutant strain. The averages and standard deviations of results of triplicate CFU plating from two independent wells for each treatment condition are shown.

Since indolamides and adamantyl ureas may have more than one target in M. tuberculosis, we next assessed whether the observed synergistic interactions were caused by the inhibition of MmpL3 by repeating the checkerboard REMA using an M. tuberculosis H37Rv mc^2^6206 strain harboring a missense mutation in MmpL3 (H37Rv MmpL3^L567P^) that is resistant to both series of inhibitors (MIC_NITD-304_ = 0.25 μg/ml; MIC_NITD-349_ = 0.5 μg/ml; MIC_AU1235_ = >0.4 μg/ml; MIC_AU36_ = >2 μg/ml). All previously observed synergistic interactions against the wild-type M. tuberculosis parent strain were lost in the mutant (Fig. 2), thereby indicating that the inhibition of MmpL3 is required for drug synergism to occur. These findings are consistent with our recent observations showing that Mycobacterium smegmatis mutants with reduced MmpL3 activity tend to be hypersusceptible to RIF, AMP, and MRP (12).

A recent study performed with indolamide inhibitors showed that these compounds synergize with RIF both *in vitro* and in an acute mouse model of TB infection (2). Likewise, combination studies performed with SQ109, another compound under development whose pleiotropic effects on actively replicating and nonreplicating M. tuberculosis include the inhibition of MmpL3 (13, 14), pointed to synergistic interactions with CFZ, BDQ, and RIF both *in vitro* and in a macrophage intracellular killing assay (5). Furthermore, replacing EMB with SQ109 improved the efficacy of the standard treatment regimens (INH, RIF, and EMB with and without pyrazinamide) in a mouse model of chronic TB (5, 15). The findings reported here indicate that synergistic interactions, not only with RIF but also with CFZ, BDQ, and β-lactams, are probably a hallmark of MmpL3 inhibitors. Possible reasons for this synergism include the increased penetration of test compounds caused by alterations in the assembling of the outer membrane, the increased stress imposed by the concomitant inhibition of two essential cell envelope biosynthetic processes (mycolic acid export and peptidoglycan biosynthesis) in the case of β-lactams, and the deleterious effect of combining MmpL3 inhibitors with agents affecting energy metabolism (CFZ and BDQ) (14, 16–20). RIF is a cornerstone of TB multidrug therapy and is required for the clearance of persister cells. RIF, however, is a potent inducer of cytochrome P-450 enzymes and the P-glycoprotein transport system and reduces the efficacy of the protease inhibitors used in the treatment of HIV (21). BDQ, CFZ, and most other second-line drugs, on the other hand, suffer from toxicity issues that reduce their clinical relevance (22). Finally, one of the primary limitations of using β-lactams therapeutically relates to the difficulty of achieving sufficient drug exposure (23). By allowing the dosages of all of these drugs to be reduced, MmpL3 inhibitors, when used in combination, may have the potential to significantly improve the tolerability and efficacy of the drug regimens used in the treatment of drug-susceptible and multidrug-resistant TB infections and of TB/HIV coinfections. These considerations and the potential that MmpL3 inhibitors have to reduce TB treatment duration (1) provide a strong incentive to further evaluate combination regimens containing MmpL3 inhibitors in animal models of TB and nontuberculous mycobacterial infections.
